# The who and the where: Attention to identities and locations in groups

**DOI:** 10.3758/s13414-024-02879-6

**Published:** 2024-05-09

**Authors:** Helen L. Ma, Ralph S. Redden, Dana A. Hayward

**Affiliations:** 1 Department of Psychology, Edmonton, Canada; 2 Neuroscience and Mental Health Institute, Edmonton, Canada; 3 Women and Children’s Health Research Institute, Edmonton, Canada

**Keywords:** Spatial attention, Social attention, Small groups, Individual differences, Gaze-facilitated responses

## Abstract

While it is widely accepted that the single gaze of another person elicits shifts of attention, there is limited work on the effects of multiple gazes on attention, despite real-world social cues often occurring in groups. Further, less is known regarding the role of unequal reliability of varying social and nonsocial information on attention. We addressed these gaps by employing a variant of the gaze cueing paradigm, simultaneously presenting participants with three faces. Block-wise, we manipulated whether one face (*Identity* condition) or one location (*Location* condition) contained a gaze cue entirely predictive of target location; all other cues were uninformative. Across trials, we manipulated the number of valid cues (number of faces gazing at target). We examined whether these two types of information (*Identity* vs. *Location*) were learned at a similar rate by statistically modelling cueing effects by trial count. Preregistered analyses returned no evidence for an interaction between condition, number of valid faces, and presence of the predictive element, indicating type of information did not affect participants’ ability to employ the predictive element to alter behaviour. Exploratory analyses demonstrated (i) response times (RT) decreased faster across trials for the Identity compared with Location condition, with greater decreases when the predictive element was present versus absent, (ii) RTs decreased across trials for the Location condition only when it was completed first, and (iii) social competence altered RTs across conditions and trial number. Our work demonstrates a nuanced relationship between cue utility, condition type, and social competence on group cueing.

**Supplementary information:**

The online version contains supplementary material available at 10.3758/s13414-024-02879-6.

## Introduction

Attention is generally thought of as a mechanism which allows people to have increased or focused awareness of attended-to stimuli (Knudsen, 2007). Attention in the real world allows individuals to take in and filter information from the environment. Evolutionarily, attentional mechanisms have developed to highlight the most prioritized and important information (Cosmides & Tooby, 2013). As such, branches of cognitive research such as social and value-driven attention argue that attention is unequally given toward social stimuli and previously rewarded stimuli, as they also confer evolutionary importance (Emery, 2000; Wang et al., 2013). In real life, there are numerous examples that come to mind of unequal attention. For example, we may be more inclined to pay attention to the gaze of our friend (perhaps signaling the unfortunate appearance of an ex) as compared with the gaze of a stranger. Perhaps we are also more likely to attend to a presenter at a meeting, compared with fellow members of the audience. Likewise, we may attend to certain nonsocial characteristics more than others. We might keep our eyes trained on the door in a waiting room, or slightly above the horizon when approaching an intersection in anticipation of a traffic light. Simple life experiences indicate attention is indeed often distributed unequally, with some stimuli receiving more attention than others. We used a modified cueing task to explore how individuals glean important information from the environment, and whether the nature of the content providing the information (social or nonsocial) matters for subsequent allocation of attention.

### Attention in the lab

In the lab, attention is frequently studied through the use of simple tasks such as the widely used cueing task (Posner, 1980). The cueing task has been employed for decades in the study of attention, is notably well-regarded, and often used to answer questions regarding the allocation and orientation of attention (Driver et al., 1999; Friesen & Kingstone, 1998; Hayward & Ristic, 2013a; Langton & Bruce, 1999; Posner, 1980; Ristic & Kingstone, 2006). The earliest versions of the cueing task employed a simple directional cue, namely a central arrow or peripheral abrupt onsets, which preceded an oncoming target after a variable time delay. Importantly, the cue could either indicate the location the target will appear (valid trial) or indicate a different location (invalid trial), and the likelihood of the cue accurately predicting the location of the target can also be manipulated, such that a cue could be irrelevant to the task (i.e., indicate the location of a target at chance level), or highly predictive of the upcoming target’s location (i.e., 100% predictive). Participants are asked to respond as fast and accurately as possible to identify the target. The logic of the task centers around the propensity of a cue to shift one’s attention; if attention is shifted in the direction of the cue, then participants will respond faster to validly-cued targets as compared with invalidly-cued targets, which is known as the cueing effect. This foundational paradigm reveals attention shifts to simple, nonsocial cues. However, information sources in the real world can be much more diverse than the simple cues in traditional cueing tasks (Driver et al., 1999; Friesen & Kingstone, 1998; Hayward et al., 2017; Langton & Bruce, 1999; Posner, 1980; Ristic & Kingstone, 2005; Ristic et al., 2006).

To reflect the complexity of information sources in the real world (social and nonsocial), the cueing task framework can be manipulated in a number of ways, including the timing between the appearance of the cue and the target, the type of cue being employed, and the predictiveness of the cue, all with the aim of answering different questions regarding the allocation of attention. Research on the time course of attention deployment has been conducted by manipulating the timing between the cue and target, with researchers suggesting that cueing effects after a short cue–target delay (i.e., stimulus-onset asynchrony [SOA]) indicates attention has been engaged quickly by the cue, and cueing effects for longer SOAs (typically 1,000 ms) indicates attention has been sustained in the direction of the cue for some time. Of note, the typical profile of attention for arrow cues is both an early and sustained cueing effect with faster responses to valid targets for SOAs ranging from 100 ms to at least 1,000 ms. In the late 1990s, the cueing task was modified by four independent groups to investigate social attention, by using a central face with averted gaze as the cue (Driver et al., 1999; Friesen & Kingstone, 1998; Hietanen, 1999; Langton & Bruce, 1999). Paradigmatic results indicate that the gaze direction of the face can shift attention, even when the gaze cue is not predictive (i.e., looks at the target at chance level; Friesen & Kingstone, 1998), or even counterpredictive (i.e., looks at the target on between 8% and 20% of trials; Driver et al., 1999; Hayward & Ristic, 2013b) of the location of an upcoming target. Ensuing work has looked at a variety of task characteristics, including SOA, on attention in the gaze cueing task (McKay et al., 2021), and found robust gaze cueing effects for short SOAs up to 800 ms. Overall, it is evident that the cueing task is a useful tool to explore different factors of attention allocation and deployment.

While the cueing task is an excellent tool to study spatial attention, by presenting a single cue on-screen, researchers are in essence pre-selecting where participants should allocate their attention (e.g., Pereira et al., 2020). This then calls into question whether spatial attention would still be allocated in the direction of the cue if competing items were also on-screen. This is an important advance: Providing a selection of items with which to attend more closely mimics the real world, as there are a myriad of items to attend to at any given moment. Thus, it is imperative to consider not only orienting one’s attention based on a pre-selected item, but also what and where participants choose to attend in the first place.

### Attention to groups on-screen in the lab

Recent work has begun addressing the issue of preselection by adapting the singular gaze cueing task to investigate group-based attention, presenting multiple gaze cues simultaneously (Capozzi et al., 2018, 2021a, b; Sun et al., 2017, 2020; Wang et al., 2019). For instance in one study, participants were shown a display of ten full body avatars, a combination of which cued the location of an oncoming target (from zero valid cues to ten valid cues), and participants were asked to identify the location or identity of a target on screen (i.e., localization and discrimination tasks respectively; Sun et al., 2017). Another two-experiment study was run, where participants were shown a display of three or five social cues (bodies or heads), a combination of which cued the location of an oncoming target (from zero valid cues to three or five valid cues), and participants were asked discriminate the target on-screen (Capozzi et al., 2018). In small groups of three it appears that a “quorum-like” rule applies, such that only one of the three faces needs to gaze toward the target in order to elicit a gaze-facilitated response (Capozzi et al., 2018, 2021a; Sun et al., 2020). In larger groups of five or ten cues, a majority rule applies, where at least three (of five) or six (of 10) faces gazing toward the target is required to elicit a gaze-facilitated response (Capozzi et al., 2018; Sun et al., 2017). In both cases, the studies investigated and established simple guidelines for eliciting gaze-facilitated responses.

However, in the real world certain people are more trustworthy than others, and we may therefore be more inclined to follow their gaze (i.e., friend’s gaze versus stranger’s). Indeed, in prototypical cueing studies, cue predictiveness is often linked to social characteristics such as trustworthiness. Studies have found that faces who consistently look toward the location of an upcoming target are rated as more trustworthy than faces who consistently look away from the location of an upcoming target (Bayliss & Tipper, 2006; Manssuer et al., 2015, 2016; Strachan et al., 2016; Strachan & Tipper, 2017). Other methods to evoke trustworthiness, such as providing a priming description of the face prior to a cueing task, have also been found to elicit larger gaze cueing effects as compared with faces who are primed with untrustworthy characteristics (Süßenbach & Schönbrodt, 2014). These results indicate that “trustworthiness” (or predictiveness) can bias attention allocation. As previous work assessing group-level orienting of attention employed non-predictive gaze cues, equating any perceived trustworthiness, it remains unclear how characteristics such as differences in trustworthiness manipulated via cue predictiveness affect the allocation of attention in a group context.

Furthermore, in the real world we allocate attention not only by social factors (i.e., trustworthy vs. not trustworthy), but also by so-called “nonsocial” factors such as which location we believe will provide us with the most useful information (i.e., attending to the door in a waiting room). Indeed, findings from contextual cueing studies suggest that nonsocial factors can also bias our attention (Chun, 2000; Chun & Jiang, 1998). Specifically, in contextual cueing paradigms participants are presented with an array of items and are required to indicate the orientation of the target; the key manipulation comes from presenting half of the displays multiple times (old), while other displays are shown only once (new). Paradigmatic results show that participants respond increasingly faster to old displays, suggesting that attention can become biased toward the target location in familiar scenes, maximizing performance. Such results inform us that biases in attention allocation can also occur for nonsocial information such as location, however it’s unclear whether biases in attention for social and nonsocial information are mediated similarly (e.g., Manssuer et al., 2016; Strachan & Tipper, 2017). Employing a paradigm with multiple cues can allow us to directly explore the effect of “useful,” predictive, social cues (e.g., specific face identities) and “useful,” predictive, nonsocial cues (e.g., specific spatial locations) on spatial attention, all with the aim of determining whether certain types of predictive information are easier to learn than others.

### Attention and participant characteristics

Finally, in the lab, as in life, it is important to consider the effects of individual subject differences. This is especially true as the nature of social attention research is predicated on the interactions between a social participant and the environment. Participant characteristics, such as gender, culture, sociability, may be a source of modulations of attention (e.g., Dalmaso et al., 2020; Zhang et al., 2021a, b). A wide array of investigations has shown autistic traits to be highly related to gaze cueing, such that individuals with higher autistic traits elicit a smaller cueing effect, thought to be related to a lack of prioritization of social stimuli (Bayliss & Tipper, 2005; Hayward & Ristic, 2017; Lassalle & Itier, 2015; Rombough & Iarocci, 2012). Likewise, males and participants with individualist political perspective demonstrate a smaller gaze cueing effect as compared with females and participants with collectivist perspectives (Alwall et al., 2010; Bayliss et al., 2005; Carraro et al., 2015; Hayward & Ristic, 2017). Therefore, we collected individual differences measures, including age, gender, ethnicity, and autistic traits, in order to contribute to our understanding of how personal factors influence attention to groups.

### The current study

Taken together, it is clear that gaze-facilitated responses to a group are complex and modulated by a myriad of different factors. Additionally, attention can be unevenly distributed through our experiences with objects in our environment, whether social or nonsocial, such as the trustworthiness of a certain face or the predictiveness of a certain context. The aim of the current study was to investigate how social and nonsocial information is experienced and used to distribute attention in a group setting, and to investigate whether this learning differs for social versus nonsocial information. To do so, we manipulated predictiveness through choosing either one identity or one location on-screen to contain the 100% predictive gaze cue.

More specifically, we ran a modified gaze cueing task with three faces on-screen instead of one. We ran two conditions. In the *Identity* condition, one of the identities contained a gaze cue that was 100% predictive of the target location, regardless of which position the face appeared. Four face identities were used, but only three faces appeared on-screen each trial. This allowed for trials in which the predictive element was not used, and thus all gaze cues could be invalid. In the *Location* condition, one of the positions contained a gaze cue that was 100% predictive of the target location, regardless of which identity appeared there. Similarly, four locations were used, but only three locations contained faces each trial. This allowed for trials in which the predictive element was not used, and thus all the gaze cues could be invalid. In both conditions, the other identities and locations contained non-predictive gaze information, gazing at the target at chance level. In all cases, the cues were placed around the fixation to prevent biasing attention to a single cue location.

This design allowed us to accomplish four objectives; importantly, the Registered Report format afforded clear delineation between our hypothesis-driven and exploratory questions. First, we attempted to replicate the previous findings on group-level gaze-facilitated responses. This was done by comparing RTs as a function of the number of valid faces. Practically, it is key for the field currently to perform and disseminate replications/extensions to avoid propagation of false positives (Open Science Collaboration, 2015) and to establish boundary conditions for observed effects (i.e., the gaze cueing effect introduced above is often not seen in populations with high autism traits, e.g., Hayward & Ristic, 2017). Second, we assessed whether participants could pick up the implicit contingency between a specific identity containing predictive gaze and the location of the target, and whether we could track learning across time. This was done by comparing RTs across trials that did and did not contain the “predictive identity.”^1^ Third, we assessed whether participants could pick up the implicit contingency between a specific “predictive location” containing a face cue and the location of the target, and whether we could track learning across time. Similarly to the identity condition, we compared RTs across trials that did and did not contain the predictive location. Fourth, we directly compared the strength of orienting to predictive identities versus predictive locations across time, as the identity and location conditions have the same design, allowing for identical trial types across the two. This allowed us to answer questions regarding the salience of identity (a social characteristic) and location (a nonsocial characteristic). Theoretically, it becomes increasingly crucial to continue probing the uniqueness of social properties in driving learning and the deployment of attention. A variety of studies have seemingly demonstrated the uniqueness of social attention above the traditional attention dichotomy or other central cues (Friesen et al., 2004; Hayward & Ristic, 2013b), while others suggest otherwise (Ristic et al., 2002). Similar patterns provide evidence of similar learning and the deployment of attention for both properties, whereas distinct patterns allow us to identify key differences between social and nonsocial learning.

#### Hypotheses

While some past research has shown priming trustworthiness modulated the gaze cueing effect, with larger magnitudes of gaze cueing for trustworthy faces (Süßenbach & Schönbrodt, 2014), other research manipulating the predictiveness of a single face showed no modulation of the gaze cueing effect, in spite of rating the predictive faces as more trustworthy than the nonpredictive faces (Bayliss & Tipper, 2006). Regardless of the mixed findings, we hypothesized that we would see modulated gaze-facilitated responses in the *Identity* condition. Logically, the predictive identity would elicit increased feelings of trustworthiness (Bayliss & Tipper, 2006) and, in turn, this should increase the gaze cueing effect for that identity (Süßenbach & Schönbrodt, 2014). Therefore, the predictive face would be more likely to elicit gaze-facilitated responses, even when the other two faces look in the other direction. Though little work has been done on the effect of predictiveness of locations, related research on contextual cueing has shown participants to be able to direct attention more effectively to displays learned through location information (Chun, 2000; Chun & Jiang, 1998). Further, spatial priority has shown that participants prioritize areas associated with greater reward (Chelazzi et al., 2014; McCoy & Theeuwes, 2018). Therefore, we should also observe modulated gaze-facilitated responses in the *Location* condition. This study was exploratory in its comparison of the salience of a social (identity) and nonsocial (location) characteristic in driving the deployment of attention. Previous research and real-life experience may influence one to consider identity as more closely linked with trustworthiness and therefore more likely to prioritize attention, however attending to faces does not necessarily equate to picking up on the contingency between gaze direction and target location. Thus, we had no firm hypotheses for our fourth objective. Finally, we predicted that greater social functioning, as measured by the Autism-Spectrum Quotient questionnaire (Baron-Cohen et al., 2001), will be associated with larger and earlier gaze cueing effects in the social *Identity*, but not nonsocial *Location,* condition (Hudson et al., 2012).

## Method

### Participants

A total of 213 observers completed the experiment. 8 were excluded due to technical errors wherein they only experienced one of the two conditions (Location Only: *N* = 6; Identity Only: *N* = 2). 14 were excluded for localizing rather than responding to the identity of the target (viz, accuracy <60%). Moreover, an additional 23 were excluded due to accuracy below our inclusion threshold (<90%).

The final sample size was 168 (117 female, 47 male, two nonbinary, two prefer not to answer; mean age = 19.84 years, *SD* = 2.15). Sample size was based on an analytic decision criterion outlined in the Results section, under “Filtering and Exclusion Criteria.” All experimental procedures were approved by the University of Alberta’s Research Ethics Office (Pro00100812). Informed consent was implied by overt action for all individual participants included in the study, as the study took place online. Participants were recruited through the undergraduate research participation pool and were awarded one course credit for their participation.

### Apparatus

The study was hosted on the Digital Research Alliance of Canada server and programmed using lab.js. Participants were instructed to complete the study in a quiet environment, with minimal distractions. Participation was restricted to use of MacBook or Windows computer, using either Chrome or Firefox internet browsers, as previous research showed MacOS and Win10 with Chrome or Firefox yielded relatively small timing variability values (variance between 4.8 and 8.1 ms; Bridges et al., 2020), and this combination allowed us a relatively wide participant pool.

### Stimuli

**Fig. 1:**
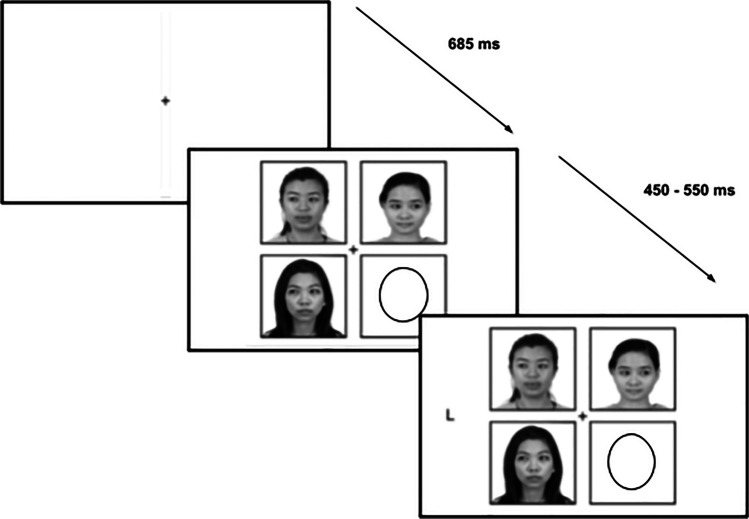
Display not to scale. At the beginning of each trial, a fixation screen was shown for 685 ms. Next, the display screen was presented. After a time interval jittered between 450 ms and 550 ms, the target letter “T” or “L” appeared on either the left or right side. The cue and target remained on the screen until a response was made or until 2,000 ms had elapsed. On each trial, three of the four face stimuli were presented in only three of the four locations. The fourth location contained an oval to balance the number of items on either side of the screen

Stimuli and an example trial sequence are illustrated in Fig. 1. There were two stimuli sets of grayscale photographs of female faces. One set of four Asian faces and one set of four Latina faces from the Chicago Face Database (CFD) were picked for their similarity in age and attractiveness (Ma et al., 2015; faces LF 203, LF 209, LF 235, LF 255, AF 228, AF 235, AF 243, AF 252; mean age = 24.05 years, mean attractiveness = 4.29 out of 5). Of note, these faces were chosen purposefully, as most investigations select White faces as stimuli, regardless of the composition of their participants, leading to limited generalizability of findings (see the recent preprint by Roberts, 2022, for an eloquent argument regarding this broader issue). As there is some evidence that the magnitude of gaze cueing is modulated by both participant and stimulus ethnicity, ethnicity information was collected from all participants, but the ethnicity relations (in-group/out-group) was not the primary focus of this investigation. All eyes were edited to be gazing to either the left or right. For each condition, one set of stimuli faces was used. The stimuli set used was counterbalanced across conditions (Asian for *Identity*, Latina for *Location*; Latina for *Identity*, Asian for *Location*). Note, sample sizes of stimuli on-screen were based on the study being presented on a MacBook Pro with a screen size 13.3-in. and a viewing distance of 24-in. A fixation cross (0.6 × 0.6 degrees of visual angle; DVA) appeared in the center of the screen for the duration of the trial. The capital letters “T” or “L” acted as targets (1.2 × 1.2 DVA), offset by 9.5 DVA to the left or right of fixation on the horizontal midline of the screen). The faces (3.6 × 3.6 DVA) were offset 4.8 DVA on the horizontal axis and 4.1 DVA on the vertical axis. The direction of gaze in such a display was demonstrated to be discernible through a pilot study (see Supplemental Materials 1).

### Design

Between subjects, we altered the predictiveness of either the face identities in the *Identity* condition (chance level to use any one face as predictive is 12.5%, each face was used between 8.3% and 14.3% across subjects) , or the predictiveness of a location on screen in the *Location* condition (chance level to use any one location as predictive is 25%, each location was used between 22.6% and 27.4% across subjects). We employed a within-subjects design, such that participants completed both the *Identity* and *Location* condition in separate blocks, with the order of blocks counterbalanced across participants. In both blocks the same stimulus configuration was used (Fig. 1), wherein faces appeared in three of four possible locations arranged in a square around central fixation. The fourth location was populated with an oval shape to balance the number of items presented on either side of fixation.

#### Identity

In the *Identity* condition, we used one set of four faces containing all Asian or Latina faces (Fig. 1). The locations of the faces were randomized across trials. Of the four candidate faces, one was predictive 100% of the time, meaning that regardless of which location it appeared, it would always gaze toward the location in which the target appeared. This created seven possible combinations of face cues. When the predictive face was present on screen there was either one face cueing the target (predictive valid), two faces cueing the target (predictive and one nonpredictive valid), or all three faces cueing the target (predictive and both nonpredictive valid). When the predictive face was absent there was either no faces cueing the target, one face cueing the target (one valid, two invalid), two faces cueing the target (two valid, one invalid), or all three faces cueing the target (three valid).

#### Location

In the *Location* condition, we used one set of four faces containing either all Asian or all Latina faces. The locations of the faces were randomized across trials. Of the four locations, one was predictive 100% of the time, which meant that regardless of the identity of the face appearing at the predictive location, it gazed toward the target. Again, this created seven possible combinations of gaze cues. When the predictive location was used (predictive present) there was either one face cueing the target (predictive valid), two faces cueing the target (predictive and one nonpredictive valid), or all three faces cueing the target (predictive and both nonpredictive valid). When the predictive location was not used (predictive absent) there was either no faces cueing the target, one face cueing the target (one valid, two invalid), two faces cueing the target (two valid, one invalid), or all three faces cueing the target (three valid).

When the predictive element was present, there were one, two, or three valid gazes toward the target (Table 1, upper portion, the predictive element is denoted “A”). When the predictive element was absent, then there were zero, one, two, or three valid gazes toward the target (Table 1, lower portion). For each condition, participants completed two blocks of 96 trials. When the predictive element was present, there were 12 combinations of gaze direction, target location, and target type. When the predictive element was absent, there were eight combinations of gaze direction and target location. To obtain the 96 trials, the former was repeated 4 times, and the latter was repeated 6 times. This approach equated the number of times each predictive and nonpredictive element was used on-screen.

**Table 1:** Trial matrix

Face/ID combos	Number of trials/block	Gaze direction	Predictive gaze direction	Target location
If face A contains predictive gaze:				
ABC/ABD/ACD	4/face combo = 12	3 left, 0 right	left	left
	4/face combo = 12	2 left, 1 right	left	left
	4/face combo = 12		right	right
	4/face combo = 12	1 left, 2 right	left	left
	4/face combo = 12		right	right
	4/face combo = 12	0 left, 3 right	right	right
BCD (i.e., no predictive gaze)				
	6	3 left, 0 right	N/A	½ left, ½ right
	6	2 left, 1 right	N/A	½ left, ½ right
	6	1 left, 2 right	N/A	½ left, ½ right
	6	0 left, 3 right	N/A	½ left, ½ right

Predictive gaze: When the predictive element was present, it always contained a gaze cue that looked in the direction of the target (equally left and right). No predictive gaze: When the predictive element was absent, the target appeared on the left or right equally, regardless of the number of faces looking to the left or right. To equate the number of instances in which each Identity or Location appeared, each Predictive Present matrix was equivalent to 1.5x the Predictive Absent matrix (each Identity/Location Combination represented by 12 of 48 trials)

### Procedure

Each trial began with a fixation cross appearing in the center of the screen for 685 ms, followed by the appearance of the faces. After a delay of roughly 500 ms (jittered between 450 and 550 ms), the target “T” or “L” appeared to the left or the right of the faces, and participants were instructed to respond by pressing the “H” or “B” key (target assignment counterbalanced across participants) as quickly and accurately as possible. The stimuli remained on-screen until a response was made, or until 2,000 ms had elapsed.

**Fig. 2:**
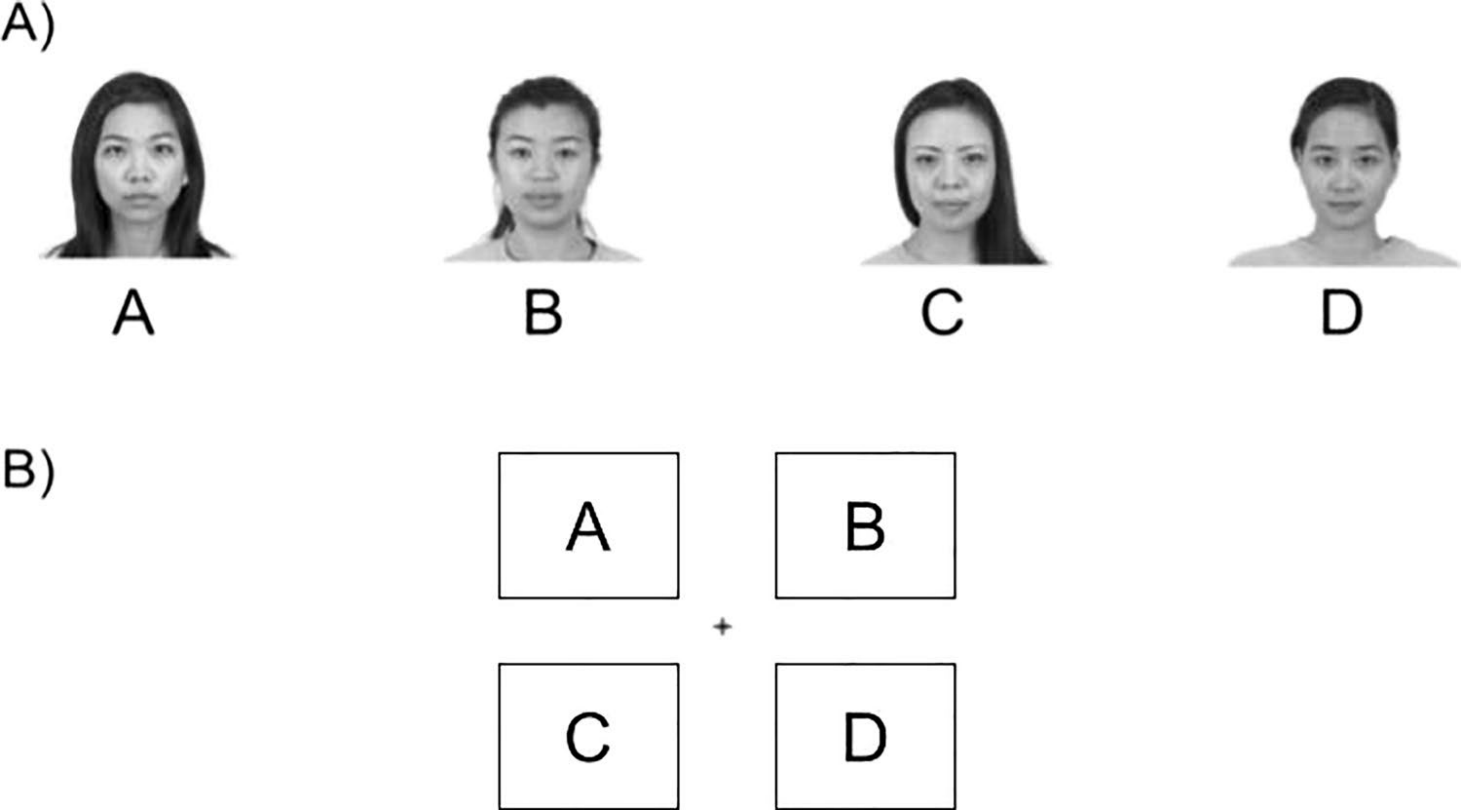
Example question schematics for the postexperiment probes, stimuli not to scale. **A)** In reference to the *Identity* condition block, participants were asked “Did you notice anything about the faces during the task?”. **B)** In reference to the *Location* condition block, participants were asked the question “Did you notice anything about the locations during the task?”

At the end of the study participants were asked two questions regarding the identities and locations used (“Did you notice anything about the faces during the task?”/“Did you notice anything about the locations during the task?”), with schematics on screen to aid in articulating a free-form response (Fig. 2). Participants were also asked what strategies they may have used (“Did you use any strategies to increase your performance on the task?”). Finally, participants completed the Autism-Spectrum Quotient questionnaire (AQ; Baron-Cohen et al., 2001). The AQ consists of 50 statements to which participants respond to on Likert-type scale (*definitely disagree* to *definitely agree*), to measure the degree of traits an individual has which are associated with the autism spectrum. It does not have diagnostic properties. This allowed us to explore if participant social characteristics relate to modulation of attention deployment by social and/or nonsocial information.

### Dissemination of materials

Raw data files, as well as the R scripts used to pre-process and analyze the data, are available on our project page hosted by the Open Science Foundation (https://osf.io/wz3n2/).

## Results

### Filtering and exclusion criteria

The relationship between RTs and error rate was used to determine RT distribution cutoffs (Christie et al., 2015; McCormick et al., 2019), providing an explicit, a priori procedure for excluding RTs prior to statistical analysis. The criterion for the faster end of the RT distribution was the fastest time in which participants were consistently responding convincingly greater than chance, which we specified a priori to be 60%. We binned RTs in intervals of 25ms to determine the 300-ms bin was the RT window at which aggregate performance across subjects was greater than 60% accuracy (66.5% accuracy; 275-ms bin = 58.8%). RTs in lower bins were excluded as anticipatory responses (1,572 trials; 2.0%).

For the slower end of the RT distribution, data were cut once a noticeable aberration in accuracy was observed as this represented an influence of non-task-related behaviours (Christie et al., 2015). The same binning procedure was used, and accuracy was compared at 25-ms bins starting at 750 ms. The upper bound of the RT distribution was based on subjective assessment of performance across bins. This cutoff was determined to be 1,075ms, which was the bin at which accuracy began to deviate from the asymptote of performance. Trials for RTs greater than 1,075 ms were excluded as too slow (3,392 trials; 4.2%). While this can be seen as less objective than the criterion for determining the lower bound, it is a data-informed decision made explicit based on the approach outlined a priori.

After these data trimming procedures, participants who did not contribute 90% of usable trials were excluded from analysis. Our a priori plan for determining sample size was as follows: Twenty-five useable participants were collected and models run. Sample size was to be incrementally increased by five participants and models rerun until we observed a minimum amount of evidence for or against the null in our primary comparison (the three-way interaction between Condition, Number of Faces, and Predictive Presence; decision criterion explained below) or until a maximum of 188 participants,^2^ whichever came first. However, after several five-observer batches of data were added and the evidence failed to move convincingly in either direction, we added larger batches and checked our decision criterion—with the intention of subsequently working backwards according to our initial plan. However, as reported in the following section, the evidence in either direction never reached our decision threshold. We subsequently terminated data collection at the end of the academic term when our subject pool closed, as there was little reason to believe the remaining few participants required to reach our a priori cutoff would make any difference in our conclusions.

### Planned analyses

Generalized linear mixed effects models were used (GLMER; lme4 R package; Bates et al., 2015) to examine the trial-by-trial relationship between predictor variables—Condition, Number of Valid Faces, Predictive Presence & AQ—and the outcome variables—RT (gaussian function).^3^ Accuracy analyses (binomial logistic link function) are included in Supplemental Materials 2; as most gaze cueing literature focuses on RT, doing so will allow us to make more in-depth comparisons, and for the sake of brevity.

Models were first run with each predictor treated as a fixed effect: Condition (Factor: Identity & Location; within-subjects), Number of Valid Faces (Continuous: 0–3; within-subjects), Predictive Presence (Factor: Present & Absent; within subjects), AQ Score (Continuous: 0–50; between subjects), with a correlated-multivariate random effect of subject on the intercept and each within-subject predictor variable. If the models did not reach convergence, to reduce complexity random effects were removed in the following order: Number of Valid Faces, Condition, & Predictive Presence.^4^ The highest-order interaction model (four-way) was run first, followed by a model with each subsequent level of lower-order interaction, ending with a model including only the main effects. Parameter estimates are reported with bootstrapped 95% confidence intervals. Likelihood ratios, with Akaike’s information criterion (AIC) corrections to account for the discrepancy of complexity between models (Akaike, 1974), were used to see if the evidence accumulated supports the effect or the null. Likelihood ratios are presented in log-base-2, so that positive values can be interpreted as evidence for the effect, and negative values as evidence for the null. The absolute value of a likelihood ratio is indicative of the confidence in the model (see Lawrence & Klein, 2013). A common heuristic for interpreting likelihood ratios is that a ratio of 8 can be considered “pretty strong” evidence, while 32 is considered “strong” (Royall, 1997). Our decision criterion for our analytic stopping rule (Lakens, 2014; see also Rouder, 2014, for a Bayesian analogue) for data collection was when there was a likelihood ratio of 20 for or against including the interaction term for the RT relationship between Condition (Identity; Location) × Number of Valid Faces (0–3) × Predictive Presence (Present; Absent). All other higher- and lower-order interactions and main effects in this model were not subject to an analytic stopping rule, and instead interpretation was made based on extant observed evidence once the a priori decision criterion threshold for our primary comparison was satisfied.

#### Four-way interactions

When examining the influence of the predictors on RT, there was no evidence to support the four-way interaction, Condition × Number of Valid Faces × Predictive Presence × AQ Score: *b* = 0.00070, 95% CI [-0.0015, 0.0016], with stronger support for the model with the interaction term dropped (AIC = −16,672) than when the term was included (AIC = −16,670), or an AIC-corrected likelihood ratio of −2.89 bits.

Therefore, the four-way model does not support our predictions, as the AIC values did not change significantly whether we kept the interaction term in or not. To follow up, we investigated whether any of the lower-order interactions revealed any significant findings.

#### Three-way interactions

To evaluate the three-way interactions for RT, we contrasted the model with all three-way interaction terms included (AIC = 16,672) with models where each term was dropped. The model performed slightly better (ΔAIC = −2) when dropping the three-way interaction term for Condition × Number of Valid Faces × Predictive Presence: *b* = −0.0025, 95% CI [-0.0080, 0.0070], or an AIC-corrected likelihood ratio of −2.89 bits. In addition, the model performed slightly better (ΔAIC = −1) when dropping the three-way interaction term for Condition × Number of Valid Faces × AQ Score: *b* = 0.0031, 95% CI [-0.0004, 0.0010], or an AIC-corrected likelihood ratio of −1.44 bits. The model performed slightly better (ΔAIC = −2) when dropping the three-way interaction term for Condition × Predictive Presence × AQ Score: *b* = −0.0001, 95% CI [-0.0014, 0.0017], or an AIC-corrected likelihood ratio of −2.89 bits. The model performed equivalently (ΔAIC = 0) when dropping the three-way interaction term for Number of Valid Faces × Predictive Presence × AQ Score: *b* = 0.0005, 95% CI [-0.0005, 0.0011], or an AIC-corrected likelihood ratio of 0 bits.

Therefore, the RT data does not support any of the three-way interactions, as the AIC values did not change significantly whether we kept any of the interaction terms in or not. To follow up, we investigated whether any of the lower-order interactions revealed any significant findings.

#### Two-way interactions

To evaluate the two-way interactions for RT, we contrasted the model with all two-way interaction terms included (AIC = 16,676) with models where each term was dropped. The model performed slightly better (ΔAIC = −3) when dropping the two-way interaction term for Condition × Number of Valid Faces: *b* = 0.0001, 95% CI [-0.0031, 0.0036], or an AIC-corrected likelihood ratio of −4.33 bits. In addition, the model performed slightly better (ΔAIC = −3) when dropping the two-way interaction term for Condition × Predictive Presence: *b* = 0.0009, 95% CI [-0.0080, 0.0083], or an AIC-corrected likelihood ratio of −4.33 bits. The model performed slightly better (ΔAIC = −3) when dropping the two-way interaction term for Condition × AQ Score: *b* = 0.0002, 95% CI [-0.0019, 0.0023], or an AIC-corrected likelihood ratio of −4.33 bits. The model performed slightly better (ΔAIC = −3) when dropping the two-way interaction term for Number of Valid Faces × Predictive Presence: *b* = 0.0011, 95% CI [-0.0027, 0.0056], or an AIC-corrected likelihood ratio of −4.33 bits. The model performed slightly better (ΔAIC = −2) when dropping the two-way interaction term for Number of Valid Faces × AQ Score: *b* = −0.0001, 95% CI [-0.0004, 0.0003], or an AIC-corrected likelihood ratio of −2.89 bits. The model performed slightly better (ΔAIC = −3) when dropping the two-way interaction term for Predictive Presence × AQ Score: *b* = 0.0002, 95% CI [-0.0006, 0.0012], or an AIC-corrected likelihood ratio of −4.33 bits.

Therefore, the RT data does not support any of the two-way interactions, as the AIC values did not change significantly whether we kept any of the interaction terms in or not. To follow up, we investigated whether any of the lower-order interactions revealed any significant findings.

#### Main effects

To evaluate the main effects for RT, we contrasted the model with all main effect terms included (AIC = -16686) with models where each term was dropped. The model performed slightly better (ΔAIC = −4) when dropping the term for Condition: *b* = −0.0026, 95% CI [-0.0150, 0.0082], or an AIC-corrected likelihood ratio of -5.77 bits. The model performed slightly better (ΔAIC = −3) when dropping the term for Number of Valid Faces: *b* = −0.0009, 95% CI [-0.0031, 0.0016], or an AIC-corrected likelihood ratio of −4.33 bits. In addition, the model performed slightly better (ΔAIC = −4) when dropping the term for Predictive Presence: *b* = 0.0008, 95% CI [-0.0041, 0.0041], or an AIC-corrected likelihood ratio of −5.77 bits. The model performed slightly better (ΔAIC = −4) when dropping the term for AQ Score: *b* = −0.0005, 95% CI [-0.0032, 0.0034], or an AIC-corrected likelihood ratio of −5.77 bits.

Therefore, the RT data does not support any of the main effects, as the AIC values did not change significantly whether we kept any of the main effects terms in or not.

### Additional exploratory comparisons

Once the criterion for our analytic stopping rule was met, additional exploratory comparisons were made. We added Trial Number and Order (Identity First; Location First) to the initial model as fixed effects, with a correlated-multivariate random effect of subject on the intercept. These six-way models were run on log(RT) and accuracy. All parameter estimates and AIC values can be found in Supplemental Materials 3, Table S1 for RT and in Table S2 for accuracy, however only those with >3 bits of evidence will be reported in text.

**Fig. 3:**
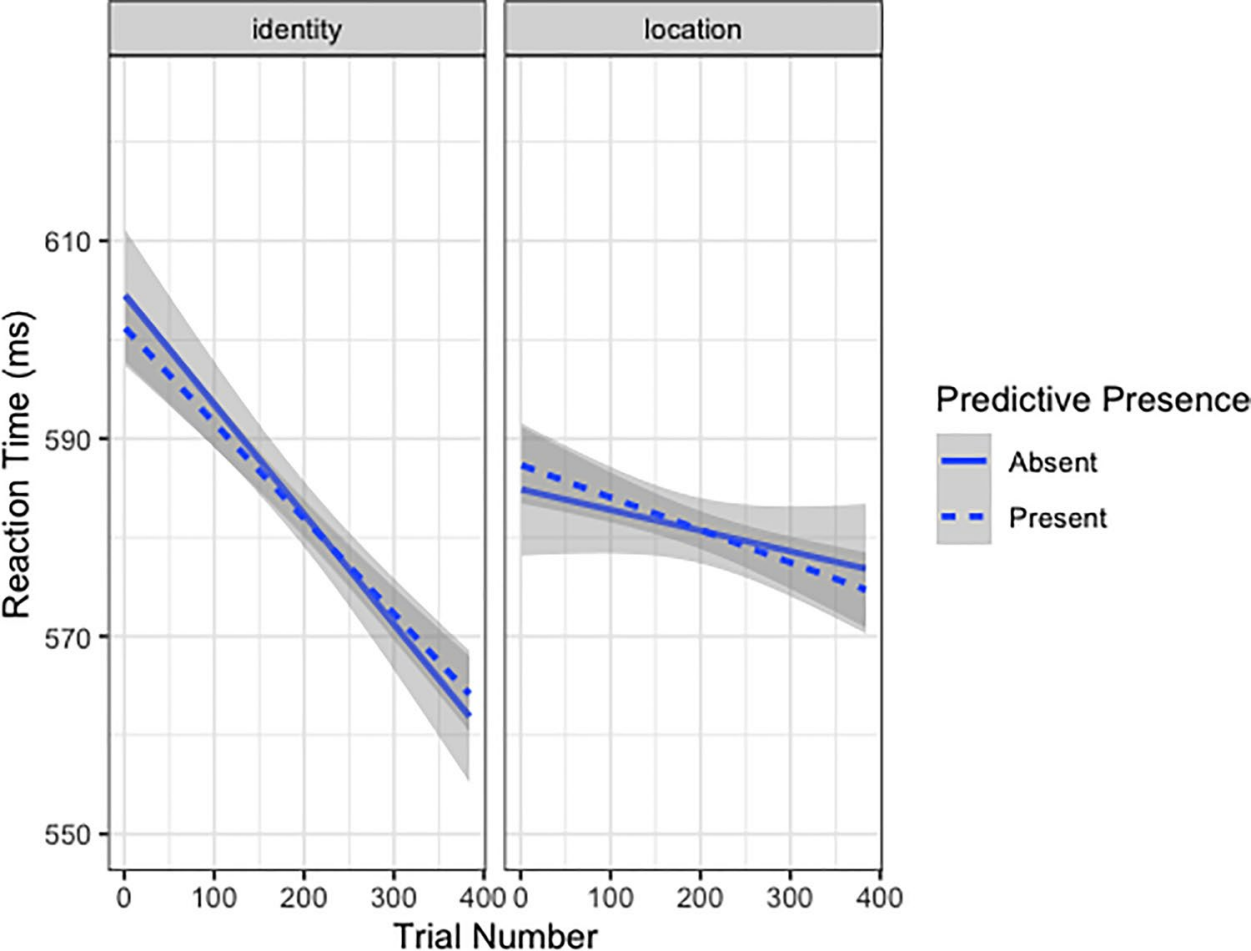
Plot of RT across trial number as a function of Condition and Predictive Presence. Solid lines represent trials where the predictive element was not used/onscreen, dashed lines represent trials where the predictive element was used/on-screen. The left side shows RT for the Identity condition, the right side shows RT for the Location condition. Confidence intervals are represented in grey. RTs decreased across trials for the Identity condition, more rapidly when the predictive element was absent compared with when it was present. RTs also decreased across trials for the Location condition, though less than in the Identity condition, with a faster change in RTs when the predictive element was present compared with when it was absent

For RT, there was sufficient evidence to support the inclusion of two three-way interactions. The model performed slightly worse (ΔAIC = +3) when dropping the term for Condition × Predictive Presence × Trial Number: *b* = −0.0002, 95% CI [-0.0003, 0.0001], or an AIC-corrected likelihood ratio of 4.33 bits (Fig. 3).

**Fig. 4:**
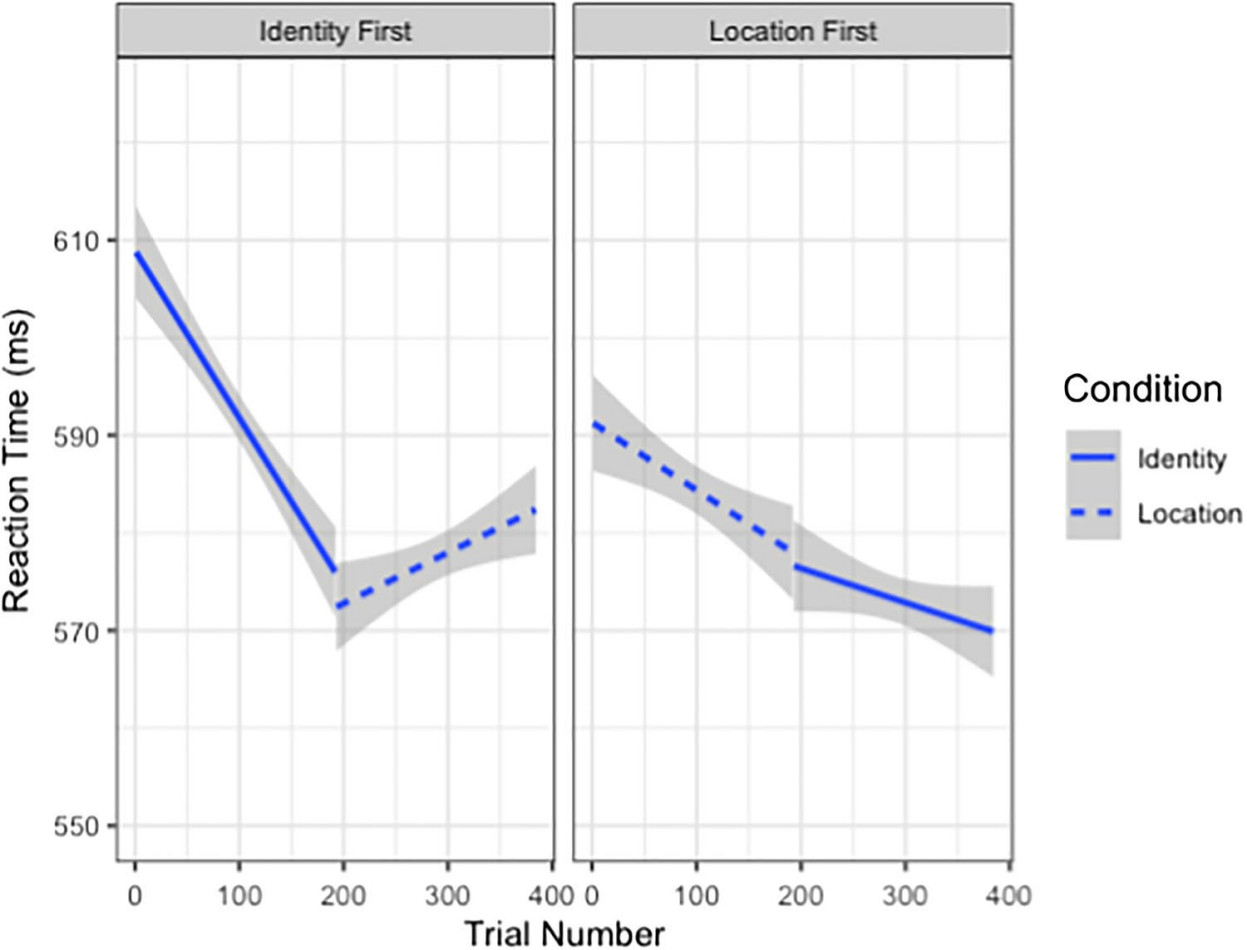
Plot of RT across trial number as a function of task order and Condition. The left side shows RT for those who completed the Identity condition first, with the first line segment (solid line) representing the Identity condition and the second line segment (dashed line) representing the Location condition. The right side shows RT for those who completed the Location condition first, with the first line segment (dashed line) representing the Location condition and the second line segment (solid line) representing the Identity condition. Confidence intervals are represented in grey. Overall, RTs decreased across trials for the Identity condition, regardless of order. RTs decreased across trials for the Location condition only when it was completed first, when completed second RTs actually increased across trials

The model performed substantially worse (ΔAIC = +45) when dropping the term for Condition × Trial Number × Order: *b* = −0.0004, 95% CI [-0.0005, -0.0003], or an AIC-corrected likelihood ratio of 64.92 bits (Fig. 4).

**Fig. 5:**
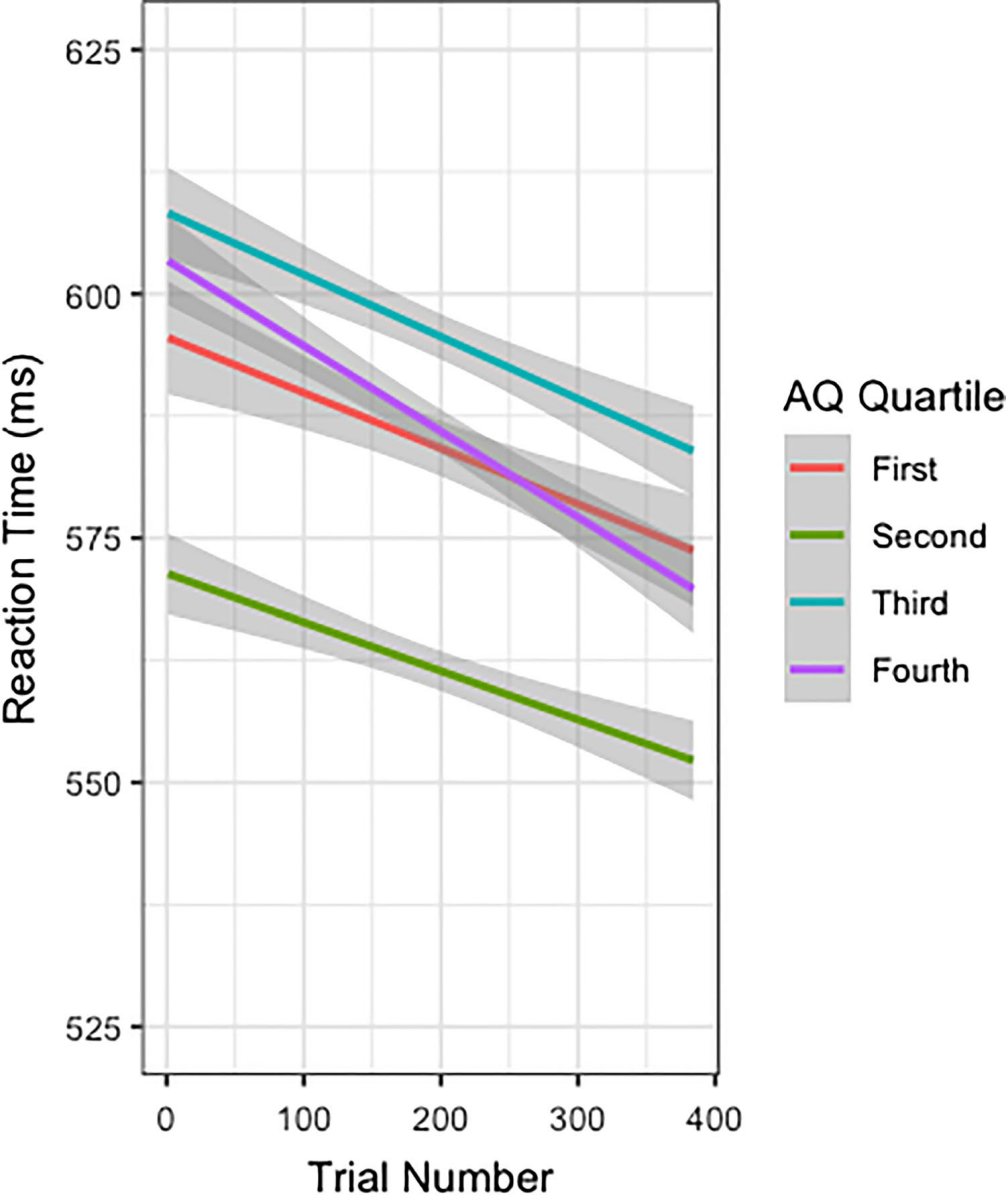
Plot of RT (ms) across trial number, divided by scores on the AQ split into four quartiles wherein the first quartile represents the lowest AQ scores (fewest Autism traits) and the fourth quantile represents the highest AQ scores (most autism traits). Score breakdown is as follows: first quartile ≤16, second quartile 17–18, third quartile 19–22, fourth quartile ≥23. Confidence intervals are represented in grey. Overall, RTs decrease across trials, with slightly different rates across trial numbers for each AQ quartile. (Colour figure online)

**Fig. 6:**
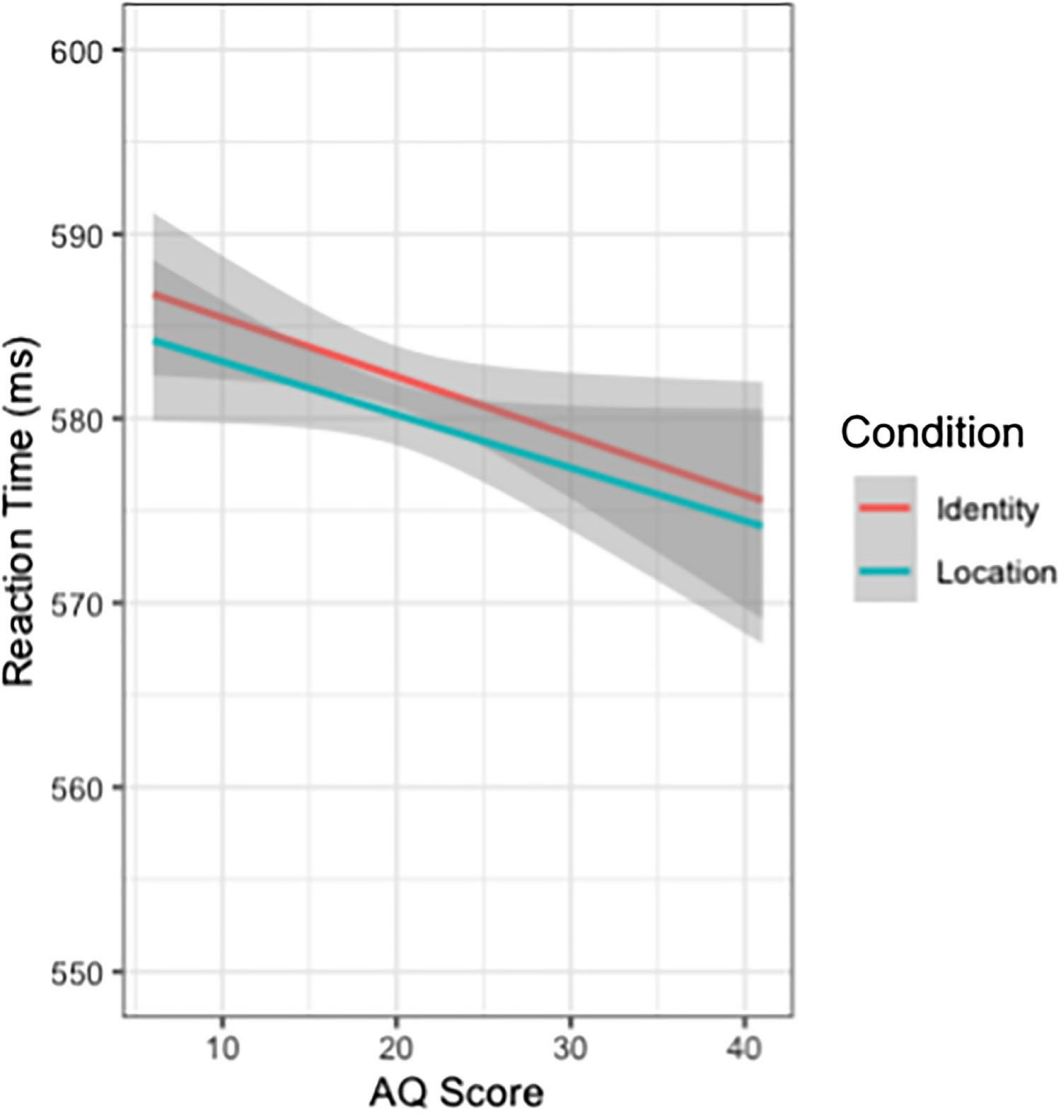
Plot of RT (ms) by AQ score, divided by condition. Overall, RTs decrease with increasing AQ scores, with slightly different rates across task type such that the decrease in RTs with increasing AQ scores is sharper for the Identity condition compared with Location. Confidence intervals are represented in grey. (Colour figure online)

There was also sufficient evidence to support the inclusion of three two-way interactions. The model performed slightly worse (ΔAIC = +4) when dropping the term for Condition × AQ: *b* = 0.0007, 95% CI [ 0.0002, 0.0015], or an AIC-corrected likelihood ratio of 5.77 bits (Fig. 6). The model performed substantially worse (ΔAIC = +24) when dropping the term for Condition × Trial Number: *b* = 0.0002, 95% CI [ 0.0001, 0.0003], or an AIC-corrected likelihood ratio of 34.62 bits. The model performed substantially worse (ΔAIC = +21) when dropping the term for AQ × Trial Number: *b* = −0.0001, 95% CI [-0.0002, -0.0000], or an AIC-corrected likelihood ratio of 30.30 bits (Fig. 5). There was sufficient evidence to support the inclusion of one main effect. The model performed substantially worse (ΔAIC = +204) when dropping the term for Trial Number: *b* = −0.0001, 95% CI [-0.001, -0.0001], or an AIC-corrected likelihood ratio of 294.31 bits.

Therefore, adding Trial Number and Order to the model specification for RT post hoc improved some of our out-of-sample deviance, as measured by ΔAIC.

## Discussion

In this study, we aimed to look at whether social and nonsocial information is differentially learned and used to orient attention in a group cueing setting. This study was conducted with four main objectives. First, to replicate early studies demonstrating a group cueing effect. Second and third, to investigate whether learning the predictiveness of social and nonsocial elements facilitates the group cueing effect. Fourth, to compare the ease of learning the predictiveness of a social versus nonsocial element. We addressed these objectives using (i) a group cueing study with three faces appearing on-screen in four possible locations, and (ii) two conditions, in which either one identity (social element) or location (nonsocial element) was 100% predictive of an upcoming target. Our results did not show any strong evidence for or against the key three-way interaction from our analysis plan, namely between Condition, Number of Valid Faces, and Predictive Presence. We did, however, find a few interactions of note during our exploratory analyses: (i) some support for an interaction between Condition, Predictive Presence, and Trial Number, (ii) strong evidence in support of an interaction between Condition, Trial Number, and Order when running the full model with six factors, and some support that social competence, as measured by the AQ, altered RTs across (iii) conditions and (iv) trial number. In addition, the main effect of Trial Number was the largest contributor to the model, accurately predicting the data for both RT and Accuracy measures, suggesting learning to some extent took place. We will discuss potential reasons for our inability to replicate the so-called group cueing effect and possible reasons for our significant findings, along with limitations.

### Null group cueing effect—What does it mean?

The most glaring inconclusive result is a lack of replication for the group cueing effect, which may arise from a number of differences, including differences due to the stimuli, due to the location of data collection, and due to the complexity of the design, which we discuss in turn.

Regarding stimulus differences, there are (at least) three key differences between the stimuli used in previous group cueing studies and the current investigation. First, most previous work used computer-generated avatar cues/stimuli, which cued the target with more than just their eye gaze (Capozzi et al., 2018, 2021a, b; Sun et al., 2017; Wang et al., 2019). For example, Sun et al. (2017) had full-bodied avatars which turned their entire body toward the possible target locations, and Capozzi et al. (2018) employed avatars from the shoulders up, which again turned toward possible target locations. In contrast, the current study used pictures of real faces with gaze alone acting as the spatial cue. Not only could this whole-body cue be more visually salient than gaze alone, but past research has also shown that the gaze cueing effect can vary based on the type of face stimulus (e.g., schematic faces vs. real faces; Hietanen & Leppanen 2003)—thus, stimulus differences could have contributed to the lack of group cueing effect we found. Along those lines, the stimuli in our study were purposefully chosen to be real faces and visually similar; all faces used were matched for race, age, gender, and attractiveness, whereas previous studies used visually distinct avatars, with different skin tones, hair colour, and shirt/pants colour. Anecdotally, participants’ free form responses seemed to indicate that many people did not consciously register the identities of the faces on screen (“the faces in Task 1 and 2 are different?”). Therefore, increasing the visual distinctiveness of the faces may have allowed participants to better learn the predictive contingencies. Second, previous studies contained an aspect of apparent movement, in that the avatars would face some central position, then a new frame would show the avatars facing a possible target location, followed by the presentation of the target. This apparent motion, which has been shown to elicit shifts of attention (Franconeri & Simons, 2003; Rees et al., 1997), did not exist in the present study as cues were already gazing toward a possible target location. This leaves open the question of whether prior “group cueing” effects were elicited through following so-called social gaze behaviour, or merely through attending to general motion cues (see also Hayward & Ristic, 2017, for a similar discussion). Third, the placement of our cues was more discrete, and potentially less “group-like” compared with previous work. This could suggest that the visually “group-like” configuration, being more naturalistic, is key in triggering the group cueing response. This is akin to work such as Böckler et al. (2014) in which two faces were used as cues, and a cueing effect was only found when the two cueing faces first established joint attention by looking at each other. This suggests that social context surrounding the cues may affect these basic tasks and processes. Together, any or all of these stimulus characteristics could have led to reductions in the saliency of the gaze cues as compared with previous work, thereby abolishing any group cueing effect in our study.

Regarding the location of data collection, prior data were collected in physical labs, often with physically present research assistants, whereas our data were collected online, likely in participant’s own place of residence with inherent distractions, and as such participants’ attention may not have been as effectively directed to the task. The motivation for the group cueing task was in part to explore the allocation of attention when the to-be-attended item was not preselected (Capozzi et al., 2018, 2021a, b; Sun et al., 2017, 2020; Wang et al., 2019). Therefore, in many ways, conducting this exploration online reflects a macroscopic attempt to reduce the experimenter’s preselection of the participant’s object of attention. In the real world, social and nonsocial factors can affect attention allocation (i.e., Bayliss & Tipper, 2006; Chun, 2000; Chun & Jiang, 1998; Manssuer et al., 2015), and the environment of the investigation may have contributed to the robustness of the effects, serving as a reminder of the importance of considering physical context as a constituent aspect of cognition (Ma et al., 2023b). The negligible effects we see in the current study could imply that face stimuli and social attention tasks commonly touted to effectively direct attention only demonstrate such efficacy in lab environments, establishing boundary conditions for such a well-known phenomenon. Finally, regarding the complexity of the design, rather than creating each person or location on-screen to be equal in “usefulness”/utility (as per previous group cueing designs), we attempted to manipulate utility to determine whether participants could learn about a specific element, social or nonsocial, and use that information to aid their performance. Perhaps the increased utility of one of the cue elements was only salient enough to affect performance (through faster RTs across trials), however not salient enough to *enable* performance *benefits*. Such was the case in Bayliss and Tipper (2006), whereby manipulating the predictiveness of different face stimuli did not modulate the cueing effect, but did affect trustworthiness ratings at the end of the study. This means that the predictiveness contingency was recognized by participants at some level, even if it did not modulate group cueing effects.

While null, these results are not uninteresting or uninformative. In fact, this lack of replication begins to establish the boundary conditions required for a group cueing effect, suggesting that perhaps group cueing is not as robust as prior work seems to indicate, with stimuli characteristics, testing location and/or complexity of the design driving social attention benefits. Additionally, the null results inform us that gaze alone is not an invariably efficient cue, particularly when it is competing for attention with other items on-screen. As noted, previous studies may have elicited a group cueing effect via incorporating general motion cues to create more salient cues than those used here, calling into question whether spatial orienting in previous group cueing studies is due to the social nature of the cue, or rather due to a more general shift of attention in response to low-level motion cues. This dovetails with previous work which questions the strong bias toward gaze alone and instead suggests underlying, basic visual components as a possible mediator (Pereira et al., 2020). Future work should continue to investigate the role that motion plays during so-called social attention tasks. While we did not replicate previous work, we feel confident in our results and statistical power, as our sample size is more than double that of previous work.

### Findings from the exploratory analyses

Though we do not see a group cueing effect, we do uncover a few significant results worth exploring. First, we found an interaction between *Condition, Predictive Presence,* and *Trial Number,* which shows participants’ performance to improve (decrease in RTs) more quickly across trials for the Identity condition compared with the Location condition, with faster performance improvements across Predictive Absent trials in the Identity condition, but faster performance improvements across Predictive Present trials in the Location condition (see Fig. 3). Therefore, the contingencies may be easier to learn in the Location condition, as we see that participants do improve in overall performance speed when the predictive element is present in the Location condition, but not in the Identity condition. Future work could include more variations of task difficulty to try and elucidate the effect of cue utility on performance. Second, we saw an interaction between *Order, Condition,* and *Trial Number,* wherein participants sped up across trials for the first condition, especially for the Identity compared with the Location condition. In comparison, for the second condition, participants *slowed down* across trials when completing the Location condition, yet we still saw some speeding up for the Identity condition (see Fig. 4). This pattern of results may initially be difficult to parse, but in fact could simply be the combination of two effects in opposing ways. This type of data pattern has been seen in previous work (i.e., Gass & Torres, 2005; Ma et al., 2023a), such as in Ma et al. (2023a) wherein researchers asked participants to play a game competitively and cooperatively, with this “game mode” counterbalanced block-wise across subjects. Their results showed two main sources of learning, from cooperating and from already completing one game mode; in one condition the two results combined together in an additive manner, whereas in the other condition the two results cancelled each other out (Ma et al., 2023a). The pattern we see in the current data may therefore reflect a differential order effect and learning effect across conditions such that (1) RT decreases at a steeper rate when the faces and contingencies are ignored, and (2) there is some learning of the contingencies in the Location condition, which paradoxically results in a shallower RT slope. As such, we can come to understand the fast, but differential, rates of improvement in the Identity condition depending on condition order. When the Identity condition is first, people are overall ignoring the faces due to condition difficulty, but when the Identity condition follows the Location condition, having to unlearn the previous contingency slows down the rate of improvement (resulting in a shallower slope). When the Location condition is first, we see a shallower RT slope across trials, but when the Location condition is second we see longer RTs across trials, as participants are attempting to learn an (easier) contingency for the first time.

The third main finding includes interactions between our measure of social competence (e.g., *AQ score*) and two task parameters; *Trial Number,* and *Condition*. The *AQ score* and *Trial Number* interaction suggests that individuals speed up faster across trials (steeper slopes) with increasing AQ scores (more autism traits; see Fig. 5). The *AQ score* and *Condition* interaction suggests that individuals speed up faster for the Identity condition compared with the Location condition (steeper slopes) with increasing AQ scores (more autism traits; see Fig. 6). Given the task, and largely null results, it is possible that the task is too difficult, and the most effective strategy is to ignore the faces on-screen. As such, individuals with high AQ scores may learn more quickly to ignore the face stimuli, more rapidly and efficiently decreasing RT. Further, in the Identity condition, the identity predictiveness information may have been more attention-grabbing for participants with fewer AQ traits, leading to slower RTs. In general, as AQ scores increase, it may be easier to ignore seemingly irrelevant social information to complete the task as quickly as possible, resulting in progressively faster RTs. This is supported by research which highlights the tendency of autistics, and people with greater autism traits, to show less bias toward social stimuli such as faces, unlike non-autistics who are easily biased toward social stimuli (Bayliss & Tipper, 2005; Hayward & Ristic, 2017; Lassalle & Itier, 2015; Rombough & Iarocci, 2012).

### Limitations

Though this study was a registered report, it is not without areas for improvement or consideration. We did use an online study, which may have allowed a broader group of participants to complete the study, but may have also resulted in inattention or divided attention as we had no control over the testing environment. Additionally, the layout of our study was different from typical in-person group environments in which group gaze shifts occur in response to a shared stimulus experience (i.e., turning toward a startling noise), though our discrete layout was somewhat reminiscent of a conference video call layout.

In sum, we conducted an adapted group cueing study to (1) replicate previous group cueing effects and (2) determine how social and nonsocial information is learned and used to orient attention in a group cueing setting. Importantly, we do not replicate the group cueing effect seen in previous work (Capozzi et al., 2018, 2021a, b; Sun et al., 2017, 2020; Wang et al., 2019), which led to our suggestion of potential boundary conditions for establishing such an effect. Additionally, we uncovered a few significant interactions, indicating that task difficulty may differentially affect performance, contingent upon condition order. This work highlights the need for research to balance replication and exploration, and the importance of not considering effects to be ubiquitous, but rather to explore potential boundaries on those effects.

## Supplementary information

Below is the link to the electronic supplementary material.

Supplementary file1 (DOCX 352 KB)

## Footnotes

1 In each case, either a specific identity or specific location contained predictive gaze information. Rather than using this longer description of the two conditions, we opted to use “predictive identity” when referring to the identity containing the predictive gaze information, and “predictive location” when referring to the location containing the predictive gaze information.

2 This is according to the guidance established by Simonsohn (2015), equivalent to 2.5 times the largest sample to date in the literature on cueing effects for groups (Capozzi et al., 2021a).

3 An exploratory log-transform was performed on the RT distribution. Based on subjective visual inspection of both distributions, the log(RT) was used as the outcome variable, as it more closely approximates the gaussian function being fit by the model.

4 The most complex model converged, so there was no need to remove any factors from the random effects term.

## Declarations

### Open science statement

As per the guidance of Simmons et al. (2012), we declare that we have reported how we will determine our sample size, all data exclusions, all manipulations, and all measures in the study. This study was conducted as a Registered Report. All project materials (data, analysis scripts, experimental code) can be found on the project page entitled: Attention to identities and locations in groups (https://osf.io/wz3n2/).

### Significance statement

This study explores the dynamics of our spatial orienting behaviours when attending to groups of people—in particular, the extent and means by which trustworthy information pertaining to task goals can be gleaned from a group. We discover that attention is not strongly shifted by social group behaviour (people looking left or right), regardless of the trustworthiness of the information provided by the Location or Identity within a group of faces. Our study suggests that highly salient social cues (i.e., including apparent motion, shifts in head direction) may be needed in a group setting to shift attention.
