# Supplementary material for: The who and the where: Attention to identities and locations in groups
Source: Atten Percept Psychophys. 2024 May 9;86(5):1816–32. doi: 10.3758/s13414-024-02879-6 (PMC11557637; doi:10.3758/s13414-024-02879-6)
Supplement: Supplementary file 1 — Supplementary file1 (DOCX 352 KB) [file 13414_2024_2879_MOESM1_ESM.docx]

**Supplemental Materials 1 - Gaze Discerning Pilot Task**

To ensure that gaze direction was discernible in our study display, we ran a gaze discerning task. The goal of this task was to check that gaze direction of all the faces would be discernible in all the used locations on screen, as the group nature of our study requires the face stimuli to be in the periphery.

**Design.** There were two versions of the gaze discerning task, one for the identity condition display (Figure S2, A) and one for the location condition display (Figure S1, B). The identity version was a 2 (gaze direction) x 3 (locations on screen) x 8 (target face identity) design. The identity version was a 2 (gaze direction) x 4 (locations on screen) x 8 (target face identity) design. This allowed us to assess the discernibility of gaze for each identity and location combination. Participants completed both the identity (48 trials /block) and location version (64 trials/block), with two blocks for both versions counterbalanced blockwise.

The procedure was identical to the gaze cueing task, with three notable exceptions. First, participants were responding to the gaze direction (left right) of a target face on screen, not an oncoming target. Preceding each trial, a display showed each of the locations on screen with a box. The location which contained the target face was indicated by a thicker border (ie., top left). This display was shown for 1000ms, following which the faces appeared. Participants then reported with key presses the gaze direction of the target face. The faces remained on screen until a response was made. Second, the non-target faces on screen had closed eyes. This was done to avoid conflicting and confusing gaze information so that participants could focus on discerning the gaze of a particular face. Third, we combined the two sets of face identities (Asian and Latina) such that any combination of the eight faces may appear on screen on any given trial.


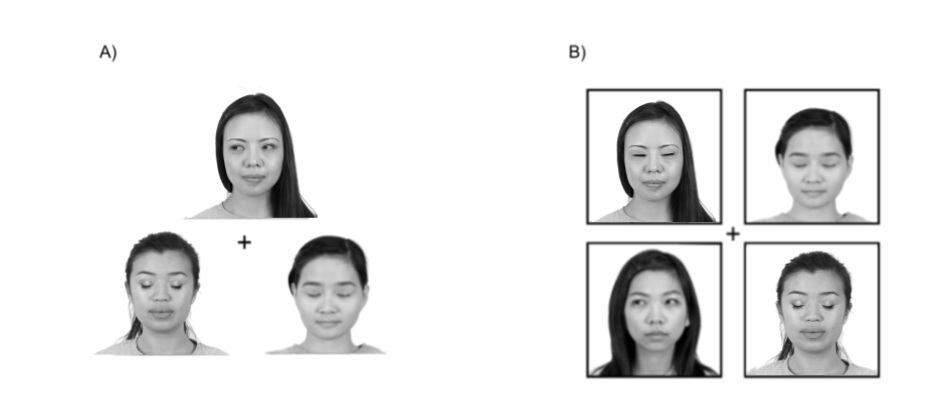


**Figure S2.** Example displays for the gaze discerning task. In task, participants saw a combination of Asian and Latina faces while the figure contains only Asian faces due to restrictions for sharing images in the CFD. A) Example identity display with three of eight possible identities used. The target face (top position) has averted gaze while the other identities have closed eyes. Participants will report the gaze direction of the target face. B) Example location display with four of eight possible identities used. The target face (bottom left position) has averted gaze while the other identities have closed eyes. Participants will report the gaze direction of the target face.

**Results**

**Processing.** In line with previous research in the field, we classed anticipatory responses as those with an RT < 150ms (0.01%), and timed out responses as those with an RT > 1500ms (11.5%).

**Analysis.** We ran a paired-samples t-test on the mean accuracy for identity as compared to the mean accuracy for location. This allowed us to compare the accuracy of gaze discerning between the identity and location manipulations.

Twenty participants completed the gaze discerning task. Two participants were excluded from the analysis due to poor overall accuracy (67.4% and 69.1% correct) relative to the remaining participants (M = 93.4%; 80.4% next lowest individual).

Mean accuracy for both conditions was quite high. In the *Identity* condition, participants discerned the gaze direction with 94.2% [CI95 +/- 2.3] accuracy, whereas in the *Location* condition, participants discerned the gaze direction with 92.8% [CI95 +/- 3.3] accuracy. A paired-samples t-test revealed no difference in accuracy of responding between the two gaze conditions: *t*(17)=1.31, *p*=0.204.

This outcome provides support that participants can accurately discern the direction of gaze at a high level. Moreover, there is no evidence that discernibility of the gaze cues differs between the *Identity* and *Location* manipulations.

**Supplemental Materials 2 - Accuracy Analyses**

**Planned Analyses**

*4-Way Interaction*

When examining the influence of the predictors on Accuracy, there was no evidence to support the four-way interaction, Condition x Number of Valid Faces x Predictive Presence x AQ Score: b = -0.0134, 95% CI = [-0.033, -0.006], with stronger support for the model with the interaction term dropped (AIC = 20216) than when the term was included (AIC = 20218), or an AIC-corrected likelihood ratio of -2.89 bits.

*3-Way Interactions*

To evaluate the three-way interactions for Accuracy, we contrasted the model with all three-way interaction terms included (AIC = 20216) with models where each term was dropped. The model performed better (ΔAIC = -1) when dropping the three-way interaction term for Condition x Number of Valid Faces x Predictive Presence: b = 0.0763, 95% CI = [-0.103, 0.227], or an AIC-corrected likelihood ratio of -1.44 bits. In addition, the model performed better (ΔAIC = -1) when dropping the three-way interaction term for Condition x Number of Valid Faces x AQ Score: b = 0.0057, 95% CI = [-0.002, 0.017], or an AIC-corrected likelihood ratio of -1.44 bits. The model performed better (ΔAIC = -2) when dropping the three-way interaction term for Condition x Predictive Presence x AQ Score: b = -0.0053, 95% CI = [-0.021, 0.016], or an AIC-corrected likelihood ratio of -2.89 bits. The model performed slightly better (ΔAIC = -1) when dropping the three-way interaction term for Number of Valid Faces x Predictive Presence x AQ Score: b = -0.0084, 95% CI = [-0.020, -0.001], or an AIC-corrected likelihood ratio of -1.44 bits.

Therefore, the accuracy data does not support any of the 3-way interactions, as the AIC values did not change significantly whether we kept any of the interaction terms in or not. To follow up, we investigated whether any of the lower-order interactions revealed any significant findings.

*2-Way Interactions*

To evaluate the two-way interactions for Accuracy, we contrasted the model with all two-way interaction terms included (AIC = 20210) with models where each term was dropped. The model performed slightly better (ΔAIC = -2) when dropping the two-way interaction term for Condition x Number of Valid Faces: b = 0.0109, 95% CI = [-0.064, 0.143], or an AIC-corrected likelihood ratio of -2.89 bits. In addition, the model performed slightly better (ΔAIC = -1) when dropping the two-way interaction term for Condition x Predictive Presence: b = -0.0892, 95% CI = [-0.290, 0.094], or an AIC-corrected likelihood ratio of -1.44 bits. The model performed slightly better (ΔAIC = -2) when dropping the two-way interaction term for Condition x AQ Score: b = -0.0024, 95% CI = [-0.018, 0.020], or an AIC-corrected likelihood ratio of -2.89 bits. The model performed slightly better (ΔAIC = -2) when dropping the two-way interaction term for Number of Valid Faces x Predictive Presence: b = -0.0121, 95% CI = [-0.119, 0.055], or an AIC-corrected likelihood ratio of -2.89 bits. The model performed slightly better (ΔAIC = -1) when dropping the two-way interaction term for Number of Valid Faces x AQ Score: b = -0.0045, 95% CI = [-0.012, 0.004], or an AIC-corrected likelihood ratio of -1.44 bits. The model performed slightly better (ΔAIC = -2) when dropping the two-way interaction term for Predictive Presence x AQ Score: b = 0.0049, 95% CI = [-0.008, 0.018], or an AIC-corrected likelihood ratio of -2.89 bits.

Therefore, the accuracy data does not support any of the 2-way interactions, as the AIC values did not change significantly whether we kept any of the interaction terms in or not. To follow up, we investigated whether any of the lower-order interactions revealed any significant findings.

*Main Effects*

To evaluate the main effects for Accuracy, we contrasted the model with all main effect terms included (AIC = 20200) with models where each term was dropped. The model performed slightly worse (ΔAIC = +1) when dropping the term for Condition: b = -0.0872, 95% CI = [-0.239, 0.045], or an AIC-corrected likelihood ratio of 1.44 bits. The model performed slightly better (ΔAIC = -2) when dropping the term for Number of Valid Faces: b = -0.0050, 95% CI = [-0.056, 0.043], or an AIC-corrected likelihood ratio of -2.89 bits. In addition, the model performed equivalently (ΔAIC = 0) when dropping the term for Predictive Presence: b = -0.0974, 95% CI = [-0.247, 0.004], or an AIC-corrected likelihood ratio of 0 bits. The model performed slightly better (ΔAIC = -1) when dropping the term for AQ Score: b = 0.0109, 95% CI = [-0.007, 0.027], or an AIC-corrected likelihood ratio of -1.44 bits.

Therefore, the accuracy data does not support any of the main effects, as the AIC values did not change significantly whether we kept any of the main effects terms in or not.

**Exploratory Analyses**

For accuracy^^[[1]](#footnote-1)^^, there was sufficient evidence to support the inclusion of one 5-way interaction. The model performed worse (ΔAIC = +11) when dropping the term for Condition x Number of Valid Faces x Predictive Presence x AQ x Order: b = -8.2189, 95% CI = [-8.589, -6.002], or an AIC-corrected likelihood ratio of 15.87 bits. There was also sufficient evidence to support the inclusion of two 4-way interactions. The model performed slightly worse (ΔAIC = +3) when dropping the term for Condition x Number of Valid Faces x Trial Number x Order: b = -1.244, 95% CI = [-2.076, -0.056], or an AIC-corrected likelihood ratio of 4.33 bits. The model performed slightly worse (ΔAIC = +4) when dropping the term for Number of Valid Faces x Predictive Presence x Trial Number x Order: b = 1.486, 95% CI = [0.925, 2.509], or an AIC-corrected likelihood ratio of 5.77 bits. There was also sufficient evidence to support the inclusion of one 2-way interaction. The model performed worse (ΔAIC = +7) when dropping the term for Trial Number x Order: b = 0.438429, 95% CI = [0.185, 0.717], or an AIC-corrected likelihood ratio of 10.10 bits. There was sufficient evidence to support the inclusion of one main effect. The model performed substantially worse (ΔAIC = +33) when dropping the term for Trial Number: b = 0.211356, 95% CI = [0.138, 0.280], or an AIC-corrected likelihood ratio of 47.61 bits.

Therefore, adding Trial Number and Order to the model specification for accuracy post-hoc improved some of our out-of-sample deviance, as measured by ΔAIC.


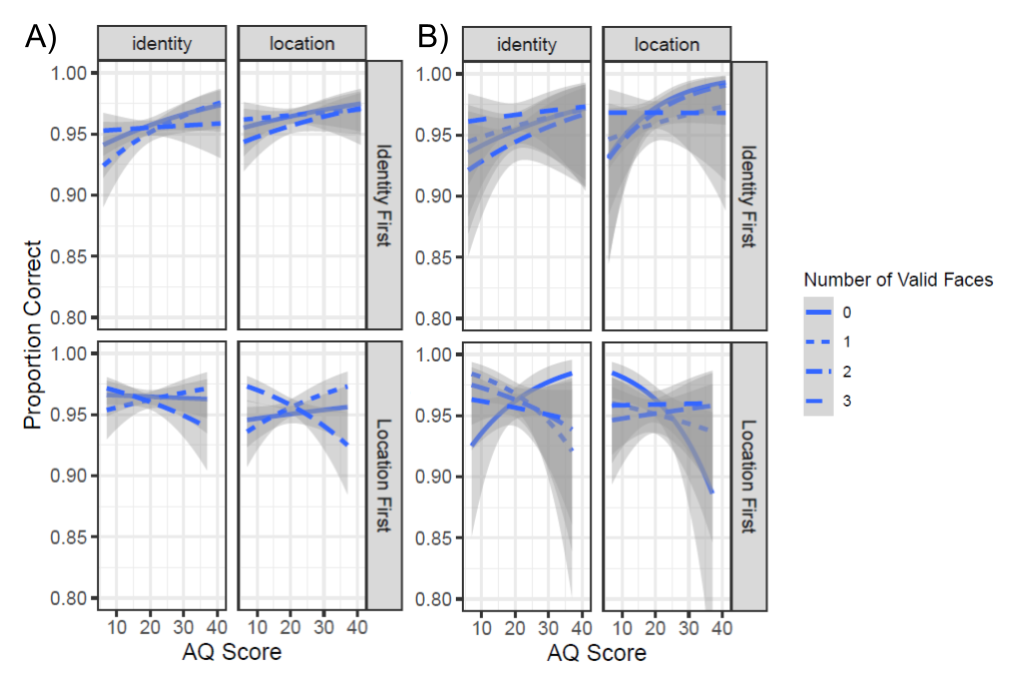


**Figure S1.** A) Plot of accuracy by AQ score when the predictive element was present, split as a function of Condition, Order, and Number of Valid Faces. B) Plot of accuracy by AQ score when the predictive element was absent, split as a function of Condition, Order, and Number of Valid Faces. Overall, accuracy decreases with both the lowest and highest AQ scores, with a greater decrease when the predictive element is absent. Changes in accuracy are even greater when there are no valid faces in the predictive absent condition. Confidence intervals are represented in grey

**Supplemental Materials 3 - Full model values**

**Table S1**

*Parameter estimates and AIC values for all response time models*

| **Model Term** | **Parameter Estimates** | **AIC-Corrected Likelihood Ratio** |
| --- | --- | --- |
| Condition x Number of Faces x Predictive Presence x ASQ x Trial Number x Order | 0.00003 | -2.885390082 |
| Condition x Number of Faces x Predictive Presence x ASQ x Trial Number | 0.00001 | -1.442695041 |
| Condition x Number of Faces x Predictive Presence x ASQ x Order | 0.0028 | 2.885390082 |
| Condition x Number of Faces x Predictive Presence x Trial Number x Order | 0.0001 | -2.885390082 |
| Condition x Number of Faces x ASQ x Trial Number x Order | 0.00001 | -2.885390082 |
| Condition x Predictive Presence x ASQ x Trial Number x Order | 0.00002 | -1.442695041 |
| Number of Faces x Predictive Presence x ASQ x Trial Number x Order | 0.0001 | 0 |
| Condition x Number of Faces x Predictive Presence x ASQ | 0.0007 | -1.442695041 |
| Condition x Number of Faces x Predictive Presence x Trial Number | 0.0001 | -1.442695041 |
| Condition x Number of Faces x Predictive Presence x Order | 0.0155 | 1.442695041 |
| Condition x Number of Faces x ASQ x Trial Number | 0.00001 | -1.442695041 |
| Condition x Number of Faces x ASQ x Order | 0.0014 | 0 |
| Condition x Number of Faces x Trial Number x Order | 0.0001 | -2.885390082 |
| Condition x Predictive Presence x ASQ x Trial Number | 0.00001 | 2.885390082 |
| Condition x Predictive Presence x ASQ x Order | 0.003 | -2.885390082 |
| Condition x Predictive Presence x Trial Number x Order | 0.0002 | -2.885390082 |
| Condition x ASQ x Trial Number x Order | 0.00001 | -1.442695041 |
| Number of Faces x Predictive Presence x ASQ x Trial Number | 0.000001 | 1.442695041 |
| Number of Faces x Predictive Presence x ASQ x Order | 0.0007 | -2.885390082 |
| Number of Faces x Predictive Presence x Trial Number x Order | 0.0001 | -1.442695041 |
| Number of Faces x ASQ x Trial Number x Order | 0.000001 | -1.442695041 |
| Predictive Presence x ASQ x Trial Number x Order | 0.0001 | 0 |
| Condition x Number of Faces x Predictive Presence | 0.0004 | -2.885390082 |
| Condition x Number of Faces x ASQ | 0.0004 | -2.885390082 |
| Condition x Number of Faces x Trial Number | 0.0001 | 0 |
| Condition x Number of Faces x Order | 0.1242 | 0 |
| Condition x Predictive Presence x ASQ | -0.0001 | -2.885390082 |
| Condition x Predictive Presence x Trial Number | -0.0002 | 4.328085123 |
| Condition x Predictive Presence x Order | -0.0348 | 2.885390082 |
| Condition x ASQ x Trial Number | 0.0001 | -1.442695041 |
| **Model Term** | **Parameter Estimates** | **AIC-Corrected Likelihood Ratio** |
| Condition x ASQ x Order | -0.0007 | -2.885390082 |
| Condition x Trial Number x Order | -0.0004 | 64.92127684 |
| Number of Faces x Predictive Presence x ASQ | 0.0005 | 0 |
| Number of Faces x Predictive Presence x Trial Number | 0.0001 | -2.885390082 |
| Number of Faces x Predictive Presence x Order | 0.0054 | 0 |
| Number of Faces x ASQ x Trial Number | 0.00001 | -1.442695041 |
| Number of Faces x ASQ x Order | 0.0004 | -1.442695041 |
| Number of Faces x Trial Number x Order | 0.00001 | -2.885390082 |
| Predictive Presence x ASQ x Trial Number | -0.00001 | -1.442695041 |
| Predictive Presence x ASQ x Order | -0.0005 | -2.885390082 |
| Predictive Presence x Trial Number x Order | -0.0001 | -2.885390082 |
| ASQ x Trial Number x Order | 0.0001 | -2.885390082 |
| Condition x Number of Faces | 0.0004 | -2.885390082 |
| Condition x Predictive Presence | 0.0014 | -2.885390082 |
| Condition x ASQ | 0.0007 | 5.770780164 |
| Condition x Trial Number | 0.0002 | 34.62468098 |
| Condition x Order | 0.0089 | 0 |
| Number of Faces x Predictive Presence | 0.0012 | -1.442695041 |
| Number of Faces x ASQ | -0.0001 | -1.442695041 |
| Number of Faces x Trial Number | 0.0001 | -1.442695041 |
| Number of Faces x Order | 0.0008 | -1.442695041 |
| Predictive Presence x ASQ | 0.0002 | -1.442695041 |
| Predictive Presence x Trial Number | -0.0001 | -2.885390082 |
| Predictive Presence x Order | 0.0004 | -2.885390082 |
| ASQ x Trial Number | -0.0001 | 30.29659586 |
| ASQ x Order | -0.0031 | -1.442695041 |
| Trial Number x Order | 0.0002 | -1.442695041 |
| Condition | -0.0022 | 0 |
| Number of Faces | -0.0008 | -1.442695041 |
| Predictive Presence | 0.0008 | -2.885390082 |
| ASQ | -0.0008 | -2.885390082 |
| Trial Number | -0.0001 | 294.3097883 |
| Order | -0.0151 | -1.442695041 |

*Note.* Parameter estimates and AIC-corrected likelihood ratios in bits for response times. Values included for all models, from the 6-way interaction to the main effects. Lines delineate each level of model complexity, for readability.

**Table S2**

*Parameter estimates and AIC values for all accuracy models*

| **Model Term** | **Parameter Estimates** | **AIC-Corrected Likelihood Ratio** |
| --- | --- | --- |
| Condition x Number of Faces x Predictive Presence x ASQ x Trial Number x Order | -1.062746 | -2.885390082 |
| Condition x Number of Faces x Predictive Presence x ASQ x Trial Number | 3.282 | 1.442695041 |
| Condition x Number of Faces x Predictive Presence x ASQ x Order | -8.2189 | 15.86964545 |
| Condition x Number of Faces x Predictive Presence x Trial Number x Order | -0.3859 | -2.885390082 |
| Condition x Number of Faces x ASQ x Trial Number x Order | 0.51324 | -2.885390082 |
| Condition x Predictive Presence x ASQ x Trial Number x Order | 4.2672 | 1.442695041 |
| Number of Faces x Predictive Presence x ASQ x Trial Number x Order | 0.30952 | -2.885390082 |
| Condition x Number of Faces x Predictive Presence x ASQ | -0.48948 | -1.442695041 |
| Condition x Number of Faces x Predictive Presence x Trial Number | 0.10579 | -1.442695041 |
| Condition x Number of Faces x Predictive Presence x Order | 0.63879 | -1.442695041 |
| Condition x Number of Faces x ASQ x Trial Number | 0.10083 | -2.885390082 |
| Condition x Number of Faces x ASQ x Order | 0.57697 | -1.442695041 |
| Condition x Number of Faces x Trial Number x Order | -1.24433 | 4.328085123 |
| Condition x Predictive Presence x ASQ x Trial Number | -1.44966 | 0 |
| Condition x Predictive Presence x ASQ x Order | 0.15214 | -2.885390082 |
| Condition x Predictive Presence x Trial Number x Order | 1.25774 | 2.885390082 |
| Condition x ASQ x Trial Number x Order | -1.03688 | -1.442695041 |
| Number of Faces x Predictive Presence x ASQ x Trial Number | 0.25837 | -1.442695041 |
| Number of Faces x Predictive Presence x ASQ x Order | -0.9059 | 0 |
| Number of Faces x Predictive Presence x Trial Number x Order | 1.48637 | 5.770780164 |
| Number of Faces x ASQ x Trial Number x Order | -0.38633 | -1.442695041 |
| Predictive Presence x ASQ x Trial Number x Order | -1.54153 | 0 |
| Condition x Number of Faces x Predictive Presence | 0.11302 | -2.885390082 |
| Condition x Number of Faces x ASQ | 0.23439 | -2.885390082 |
| Condition x Number of Faces x Trial Number | 0.53513 | 1.442695041 |
| Condition x Number of Faces x Order | 0.69579 | 2.885390082 |
| Condition x Predictive Presence x ASQ | -0.13202 | -2.885390082 |
| Condition x Predictive Presence x Trial Number | 0.20278 | -2.885390082 |
| Condition x Predictive Presence x Order | -0.0126 | -2.885390082 |
| Condition x ASQ x Trial Number | 0.12813 | -2.885390082 |
| Condition x ASQ x Order | -0.36066 | -2.885390082 |
| Condition x Trial Number x Order | 0.48014 | 1.442695041 |
| **Model Term** | **Parameter Estimates** | **AIC-Corrected Likelihood Ratio** |
| Number of Faces x Predictive Presence x ASQ | -0.33961 | -1.442695041 |
| Number of Faces x Predictive Presence x Trial Number | -0.07284 | -2.885390082 |
| Number of Faces x Predictive Presence x Order | 0.26415 | 0 |
| Number of Faces x ASQ x Trial Number | -0.05283 | -2.885390082 |
| Number of Faces x ASQ x Order | -0.18548 | -2.885390082 |
| Number of Faces x Trial Number x Order | -0.40279 | 0 |
| Predictive Presence x ASQ x Trial Number | -0.2174 | -2.885390082 |
| Predictive Presence x ASQ x Order | 0.4334 | -1.442695041 |
| Predictive Presence x Trial Number x Order | 0.4538 | -1.442695041 |
| ASQ x Trial Number x Order | 0.93653 | 1.442695041 |
| Condition x Number of Faces | 0.008294 | -2.885390082 |
| Condition x Predictive Presence | -0.094152 | -1.442695041 |
| Condition x ASQ | 0.025802 | -2.885390082 |
| Condition x Trial Number | 0.296447 | 2.885390082 |
| Condition x Order | 0.102812 | -2.885390082 |
| Number of Faces x Predictive Presence | -0.041956 | -2.885390082 |
| Number of Faces x ASQ | -0.285523 | 1.442695041 |
| Number of Faces x Trial Number | -0.089209 | -1.442695041 |
| Number of Faces x Order | 0.059268 | -2.885390082 |
| Predictive Presence x ASQ | 0.09268 | -2.885390082 |
| Predictive Presence x Trial Number | 0.01264 | -2.885390082 |
| Predictive Presence x Order | 0.045835 | -2.885390082 |
| ASQ x Trial Number | -0.072326 | -2.885390082 |
| ASQ x Order | -0.596425 | 0 |
| Trial Number x Order | 0.438429 | 10.09886529 |
| Condition | 0.009382 | -2.885390082 |
| Number of Faces | -0.021419 | -2.885390082 |
| Predictive Presence | -0.035809 | -2.885390082 |
| ASQ | 0.210931 | -1.442695041 |
| Trial Number | 0.211356 | 47.60893635 |
| Order | -0.030312 | -2.885390082 |

*Note.* Parameter estimates and AIC-corrected likelihood ratios in bits for accuracy. Values included for all models, from the 6-way interaction to the main effects. Lines delineate each level of model complexity, for readability.

1. The continuous effects (Trial Number, AQ, Number of Valid Faces) were standardized for the exploratory accuracy models, due to a failure to converge. [↑](#footnote-ref-1)
